# Roles of NF-κB Signaling in the Regulation of miRNAs Impacting on Inflammation in Cancer

**DOI:** 10.3390/biomedicines6020040

**Published:** 2018-03-29

**Authors:** Georgios S. Markopoulos, Eugenia Roupakia, Maria Tokamani, Georgia Alabasi, Raphael Sandaltzopoulos, Kenneth B. Marcu, Evangelos Kolettas

**Affiliations:** 1 Laboratory of Biology, School of Medicine, Faculty of Health Sciences, University of Ioannina, 45110 Ioannina, Greece; gmarkop@cc.uoi.gr (G.S.M.); ev.roupakia@gmail.com (E.R.); gwgw_al95@hotmail.com (G.A.); 2 Biomedical Research Division, Institute of Molecular Biology and Biotechnology, Foundation for Research and Technology, 45110 Ioannina, Greece; kenneth.marcu@stonybrook.edu; 3 Department of Molecular Biology and Genetics, Democritus University of Thrace, 68100 Alexandroupolis, Greece; tokamanimaria@hotmail.com (M.T.); rmsandal@mbg.duth.gr (R.S.); 4 Biomedical Research Foundation of the Academy of Athens, 4 Soranou Ephessiou Street, 115-27 Athens, Greece; 5 Departments of Biochemistry and Cell Biology, Microbiology and Pathology, Stony Brook University, Stony Brook, NY 11794-5215, USA; 6 Department of Biology, San Diego State University, San Diego, CA 92182-4614, USA

**Keywords:** miRNAs, NF-κB, transcriptional regulatory networks, oncogenic and tumor suppressor pathways, cancer, inflammation

## Abstract

The NF-κB family of transcription factors regulate the expression of genes encoding proteins and microRNAs (miRNA, miR) precursors that may either positively or negatively regulate a variety of biological processes such as cell cycle progression, cell survival, and cell differentiation. The NF-κB-miRNA transcriptional regulatory network has been implicated in the regulation of proinflammatory, immune, and stress-like responses. Gene regulation by miRNAs has emerged as an additional epigenetic mechanism at the post-transcriptional level. The expression of miRNAs can be regulated by specific transcription factors (TFs), including the NF-κB TF family, and vice versa. The interplay between TFs and miRNAs creates positive or negative feedback loops and also regulatory networks, which can control cell fate. In the current review, we discuss the impact of NF-κB-miRNA interplay and feedback loops and networks impacting on inflammation in cancer. We provide several paradigms of specific NF-κB-miRNA networks that can regulate inflammation linked to cancer. For example, the NF-κB-miR-146 and NF-κB-miR-155 networks fine-tune the activity, intensity, and duration of inflammation, while the NF-κB-miR-21 and NF-κB-miR-181b-1 amplifying loops link inflammation to cancer; and p53- or NF-κB-regulated miRNAs interconnect these pathways and may shift the balance to cancer development or tumor suppression. The availability of genomic data may be useful to verify and find novel interactions, and provide a catalogue of 162 miRNAs targeting and 40 miRNAs possibly regulated by NF-κB. We propose that studying active TF-miRNA transcriptional regulatory networks such as NF-κB-miRNA networks in specific cancer types can contribute to our further understanding of the regulatory interplay between inflammation and cancer, and also perhaps lead to the development of pharmacologically novel therapeutic approaches to combat cancer.

## 1. Introduction

Carcinogenesis involves the accumulation of mutations in conjunction with epigenetic changes resulting in dominant alterations in gene expression and cellular physiology. NF-κB TFs and their signaling pathways play important roles in cellular growth and viability control and are often subject to deregulation in cancer. Oncogenic driver mutations and inactivating mutations in tumor suppressor genes along with epigenetic changes in normal cells, can lead to the growth of tumor containing cells with distinct phenotypic characteristics, known as the hallmarks of cancer [1]. Cancer cells are also characterized by extensive epigenetic alterations compared to their normal counterparts, as a result of deregulated tissue-specific gene regulatory mechanisms. Elucidating the interaction between genetic and epigenetic factors in cancer onset, development, and progression is considered as a main challenge in both our understanding of cancer biology and for the development of new therapeutic approaches [2,3].

Transcriptional control of gene expression involves binding of TFs to regulatory elements in gene promoters or enhancers. NF-κBs constitute a family of TFs that influence the expression of genes involved in many physiological processes, such as cell proliferation, cell survival, cell adhesion, inflammation, and immunity. The NF-κB signaling components are aberrantly expressed and/or activated in cancer [4,5,6,7,8]. NF-κBs play a central role in the regulation of inflammatory responses at the cellular and systemic levels, and can have tumor promoting effects [9]. However, the NF-κB biology is strikingly complex and NF-κB TFs and their upstream activating signaling components can have either tumorigenic or tumor suppressor roles in cell context-dependent manner and under certain conditions [6,10].

Genes encoding ~22 bp long, small non-coding RNAs, known as microRNAs (or miRNAs or miRs) are emerging as major epigenetic regulators of cell physiology and/or pathology [11,12,13]. MiRNAs regulate gene expression at the posttranscriptional level by acting as negative regulators of mRNA translation and/or stability resulting in the suppression of translation [13,14], and play an important role in inflammatory and immune responses [15,16,17,18] and cancer [11].

In cancer, miRNAs can act as oncogenes, targeting tumor suppressor mRNAs or as tumor suppressors, targeting oncogenic mRNAs. MiRNA genes can also be mutated or epigenetically altered, and suppressed or activated by transcription factors leading to changes in their expression [11]. Importantly, the balance between oncogenic and tumor suppressor miRNAs expressed in a cell, can be a major epigenetic factor that influences cancer onset, development, and progression [11]. Hence, the specific expression of miRNAs and their interplay may tip that balance towards cell proliferation, leading to tumor expansion, or cell cycle arrest, senescence or apoptosis leading to the impairment of tumor growth [11].

The epigenetic mechanisms of TF or miRNA regulation, act at different stages of gene expression, and have some unique features but also share some similarities [19]. TFs can target and regulate the expression of specific miRNAs and, vice versa, miRNAs can target TF mRNAs. This property of TF and miRNA regulation, offers the cells an opportunity to create genomic-scale regulatory networks in which positive or negative feedback loops can act in concert to influence the epigenomic landscape of cells [20]. In the current review, we discuss the specific roles of the regulatory networks between NF-κB TFs and miRNAs and their impact on the conditions of inflammation and cancer development, as well as their interactions.

## 2. NF-κB Signaling Pathway Activation and Its Multifaceted Functional Role in Cancer and Inflammation

The NF-κB TF family members are critical regulators of pro-inflammatory/stress-like responses. There are three protein subfamilies involved in NF-κB signaling: The NF-κB TF subunit subfamily (c-Rel, p65/RelA, RelB, p105/NF-κB1, and p100/NF-κB2), the regulatory family of NF-κB inhibitors, inhibitors of κ*B* (IκBs), and the catalytic IKK (Inhibitor of NF-κB (IκB) kinase (IKK) complex) subfamily comprising the NF-κB upstream activating Ser/Thr kinases IKKα and IKKβ and a regulatory protein NEMO (NF-κB essential modulator)/IKKγ that together form a high molecular weight IKK signalosome complex, that activates NF-κB signaling in response to specific stimuli. Members of the NF-κB TF subfamily bind to DNA as hetero- or homodimers and can either activate or repress target gene transcription in different physiological contexts. Three of these NF-κB subunits (c-Rel, p65/RelA, and RelB) contain a transactivation domain (TAD), while the other two (p50 and p52) lack a TAD domain and are derived by proteolytic processing of the larger precursor proteins, p105/ NF-κB1 and p100/NF-κB2, respectively. Activation of NF-κB signaling occurs by two major pathways: the canonical NF-κB pathway and the noncanonical or alternative NF-κB pathway. In unstimulated cells, heterodimers of p65/p50 subunits, involved in canonical NF-κB signaling, are retained in the cytoplasm by IκBs. Pro-inflammatory and stress stimuli lead to NEMO-dependent activation of IKKβ by phosphorylation of Ser177/181. Activated IKKβ then phosphorylates IκBα at Ser32/36 resulting in its proteasomal degradation and the release of p65/50 heterodimer that translocates to the nucleus where it binds and regulates target gene expression. In contrast, IKKα activation, through phosphorylation of Ser176/180, by adaptive immune response stimuli, is mediated by the NF-κB inducing kinase (NIK). IKKα kinase phosphorylates NF-κB2, inducing its proteasomal processing yielding the mature p52 subunit. Active p52/RelB heterodimers translocate to the nucleus and regulate distinct NF-κB target genes [4,6,7,21,22].

NF-κB target genes encode proteins and miRNAs that regulate a wide range of biological effects that together can be categorized as stress-like, pro-inflammatory reaction programming. The NF-κB signaling pathways have pleiotropic biological effects which may be context dependent. In cancer, NF-κB can exhibit tumor promoting and tumor suppressor activities in a cell context- and tissue-dependent manner [6]. Several mouse cancer models have shown a requirement for canonical NF-κB signaling in tumor onset, development and progression [6,23,24,25,26,27,28,29,30,31,32,33,34,35,36,37].

The tumor promoting effects of NF-κB are mediated by the activities of NF-κB-regulated genes that promote cancer cell survival, proliferation, metastasis, and angiogenesis, and modify the tumor microenvironment by inducing the secretion of proinflammatory cytokines. NF-κB also promotes a cancer cell metabolic switch from oxidative phosphorylation to glycolysis (Warburg effect) by inducing the expression of glycolytic enzymes while also directly repressing mitochondrial gene expression [38,39,40,41]. Thus, NF-κBs function as tumor promoters within transformed cells, but also influence the host’s innate immune response to cancer cells by regulating functions of infiltrating lymphocytes and macrophages [22,42]. Although under physiological conditions NF-κB responses are self-limiting via the induction of negative feedback loops, such auto-regulatory loops often become deregulated in cancer cells. However, the regulatory circuitry that leads to dominant IKK/NF-κB-dependent effects in cancer is impressively complex [6,7,9,23].

### 2.1. Oncogenic Functions of NF-κB: A Link between Inflammation and Cancer

Epidemiological, clinical, genetic, and biochemical evidence obtained from cells, tissues, and mouse models indicate that NF-κB-dependent induction of pro-inflammatory cytokines are pivotal links between chronic inflammation and cancer development and progression [43,44,45,46,47,48].

Inflammation can either promote tumor growth, or it may be induced as a consequence of the tumor microenvironment leading to cancer progression [44]. Inflammation promotes cancer onset, development, and progression, and it also affects the immune surveillance and chemotherapy resistance of tumors. In addition, inflammation affects the crosstalk between infiltrating immune effector cells and tumor cells thereby linking immunity to tumor development [9,22,24,49,50,51,52].

NF-κB TFs have a central role in innate immunity, inflammation, and cancer [6,7,8,22,42,48,49,50,51,52,53,54,55]. NF-κBs induce inflammation and the secretion of inflammatory mediators enhances canonical NF-κB signaling [9], a feedback mechanism acting as tumor promoter [8,25,26,48,56], and a hallmark of cancer [1]. In chronic inflammation, canonical NF-κB that controls production of inflammatory mediators might prevent the elimination of genetically altered cells present in precancerous lesions by inhibiting their apoptosis [57]. Tumor-associated macrophages (TAMs) were shown to promote tumor growth in part by suppressing immune response to cancer cells but also by producing specific cytokines, most of which are dependent on IKKβ-mediated canonical NF-κB signaling (e.g., IL-6) that enhance tumor cell growth in vivo [9,56,58]. Canonical NF-κB also modifies the tumor microenvironment by inducing the secretion of proinflammatory cytokines such as IL-6, resulting in the activation of its responsive transcription factor STAT3 in *K-Ras*-mutant lung tumors [58]. IL-6 modifies the tumor microenvironment and promotes breast and lung cancer development and progression [58,59]. NF-κB functions in *K-Ras* oncogene transformation by suppressing immune surveillance of both innate and adaptive immune cells [60]. Moreover, canonical NF-κB pathway activation and the interplay with other signaling pathways such as those of STAT3 and p53, may affect tumor onset, development, and progression [44]. One of the critical contributing factors to the oncogenic functions of canonical NF-κB signaling is the induction of inflammation making NF-κB as the critical link between inflammation and cancer [27,28,44,45,46,48].

While the contribution of canonical NF-κB-activating IKKβ as a tumor promoter in oncogene and carcinogen-induced inflammation and non-small cell lung cancer (NSCLC) has been documented [25,30,31,34] functional studies on noncanonical NF-κB [61,62,63,64] and IKKα [65,66,67] suggest that they can act as tumor promoters or tumor suppressors and are involved in the resolution of inflammation [68,69,70,71,72], but an evolutionary conserved mechanism of action remains largely unknown. These different outcomes of canonical versus non-canonical NF-κB signaling pathways may be related to the preference of NF-κB dimers for binding to *κB* sites contained within the promoters or enhancers of target genes. Sensing the differences within *κB* sites, NF-κB dimers modulate physiological programs by activating, repressing, and altering the expression of effector genes [73,74,75].

A crosstalk between canonical and noncanonical NF-κB signaling pathways has also been shown. It was shown that NF-κB2 [76,77] and RelB [78] gene expression is induced by canοnical NF-κB signaling. RelA/p65 suppresses RelB activity in response to TNFα and induces selective NF-κB target gene expression [79]. It was also shown that TNFα-induced canonical NF-κB signaling upregulates RelB expression that inhibits both basal and non-canonical NF-κB-dependent CXCL12 expression [80]. NIK which activates noncanonical NF-κB signaling may also contribute to the activation of canonical NF-κB [81]. While IKKα activates noncanonical NF-κB signaling, evidence show that it also inhibits the canonical NF-κB pathway [82,83,84]. It was also shown that nuclear IKKα is required for p65 DNA binding in a gene-specific manner [85].

NF-κB TFs are often deregulated and constitutively activated in many different types of cancer [4,6,53], leading to the development of different hallmarks of cancer [1]. NF-κB’s function as a tumor promoter is also due to its role in driving cell proliferation and protecting cells from cell death under stress conditions by regulating the expression and activity of target genes involved in cell cycle progression and apoptosis [5,6,7,9,49,86,87,88]. Canonical NF-κB was shown to activate genes involved in cell cycle progression such as CcnD1 [5,86,89,90], E2F1 [5,86], and several E2F target genes [5] and the mitotic checkpoint Ser/Thr-protein kinase BUB1 [34]. It was also shown to suppress genes involved in apoptosis such as FOXO3a, leading to increased cell survival [4,21,91,92]. In keeping with this, miR-155, a canonical NF-κB regulated miRNA, was identified as a negative regulator of FOXO3a leading to increased gefitinib resistance and lung cancer stemness in vitro and in vivo [92]. NF-κB also suppresses the expression of c-Jun N-terminal kinase (JNK) via Gadd45β and blocks apoptosis [93,94]. Canonical NF-κB also contributes to chemoresistance of tumor cells such as leukemic cells, in part through its ability to induce p21^waf1/cip1^ [95,96] and p27^Kip1^ [97].

NF-κB targets that play an important role in cancer progression are those involved in epithelial-to-mesenchymal cell transition (EMT), such as Snail, Twist, matrix metalloproteinases (MMPs) and cell adhesion molecules that promote metastasis, and pro-angiogenic genes such as Vascular Endothelial Growth Factor (VEGF), stimulating tumour neovascularization [8,48,98,99,100]. Canonical NF-κB also regulates the expression of matrix metalloproteinases involved in tissue remodeling, inflammatory diseases and cancer [101,102,103,104,105]. In addition, Timp1 (tissue inhibitor of metalloproteinase 1), was identified as a NF-κB target gene that contributes to mouse lung tumor growth [34], and it is highly expressed, and correlates with NF-κB activation in advanced lung-cancer patients with poor prognosis [106,107]. NF-κB is also a critical transcriptional regulator of HIF1α, and IKKβ-mediated canonical NF-κB activation is required for the hypoxia-induced accumulation of HIF1α and the expression of HIF1α target genes [108,109]. Several lines of evidence suggest a bi-directional crosstalk between NF-κB and HIF pathways, with the latter also contributing to inflammatory responses and cancer [109,110,111,112,113].

In physiological conditions, NF-κB activity is tightly regulated and inhibited after a short period of time through negative feedback loops [4]. Based on this concept, aberrant NF-κB signaling activation leading to chronic inflammation and increased cell proliferation and survival are additional factors contributing to the oncogenic function of NF-κB [6,23,47,48].

### 2.2. Tumor Suppressor Function of NF-κB

NF-κBs can also suppress tumor growth under certain conditions, a functional role dependent on the presence and crosstalk with tumor-suppressor-proteins, such as p53, which modulate NF-κB activity in cancer. These tumor suppressive functions of NF-κB are due to NF-κB-dependent activation of gene expression that can lead to the inhibition of cancer cell cycle progression and proliferation, apoptosis, suppression of cell invasion, and metastasis [6,55,114,115].

The tumor suppressive functions of canonical NF-κB may be due to the modulation of NF-κB activity by tumor suppressors such as p53 [6,61,116,117,118] or due to alterations in the phosphorylation status of NF-κB subunits [6,119,120,121] suppressing NF-κB’s ability to induce the expression of genes that are associated with tumor growth and survival. Canonical NF-κB can also inhibit tumor growth by inducing the expression of tumor suppressors such as Bach2 induced in B-cells by c-Rel or RelA suggesting a tumor suppressive function of c-Rel in B-cell lymphoma [114]. *c-Myc* overexpression was shown to sensitize cells to NF-κB-induced apoptosis, and persistent inactivity of NF-κB signaling was shown to be a prerequisite for *c-myc*-mediated lymphomagenesis [122].

The tumor suppressive functions of canonical NF-κB may also be attributed to an attenuated inflammatory response.NF-κB p50 subunit functions as a transcriptional regulator either as a heterodimer with NF-κB subunits RelA, c-Rel, and RelB, or as a p50 homodimer. p50 heterodimers induce gene expression and are critical in inflammatory responses, while p50 homodimers generally act as transcriptional repressors [7,55,123]. The p50homodimer has an important function as suppressor of inflammation through repressing proinflammatory gene expression while enhancing the expression of anti-inflammatory genes [55,124,125]. *Nfkb1*(p105/p50)^-/-^ mice display increased inflammation and susceptibility to DNA damaging agents, leading to cancer including lymphomas and liver cancer, and an ageing phenotype [55,126,127]. Reduced levels of p50 were observed in human tumor tissues from head and neck and glioblastoma cancers; and these results were further supported by xenograft models of human glioblastoma and breast cancer cell lines in mice [128].

The tumor suppressive functions of noncanonical NF-κB may be attributed to a reduced inflammatory response and oxidative stress [29,52,65,67,70,129,130]. For example, enforced expression of a kinase-dead IKKα mutant protein in mice led to spontaneous lung squamous cell cancer (SCC) development and the recruitment of TAMs, suggesting a tumor suppressor role for IKKα in lung SCC [65,130]. IKKα loss has also been reported to promote *K-Ras*-initiated NSCLC development through a redox regulatory pathway involving ROS accumulation [67].

Emerging evidence suggests that the tumor promoting or suppressive functions of NF-κB, in a cell- and tissue-dependent context may also be determined by miRNAs and their targets. Thus the IKK/NF-κB-miRNA transcriptional regulatory network may play a critical role in inflammation impacting on cancer [11].

## 3. MiRNAs: Epigenetic Regulators in Inflammation and Cancer

MiRNAs regulate gene expression at the post-transcriptional level acting as negative regulators of mRNA translation and/or stability by binding to complementary sequences in the 3′ untranslated region (3′ UTR) of their target mRNAs. Individual miRNAs may target several different mRNAs to inhibit their translation into polypeptides, partly because target sites on an mRNA require only partial base complementarity with their corresponding miRNAs. In cases of perfect complementarity, cleavage of the target mRNA is induced. Moreover, individual mRNAs may contain multiple binding sites for different miRNAs, resulting in complex regulatory networks. Conversely, binding sites for a specific miRNA may be limited to few mRNAs, while others may target a larger number of mRNAs. Hence, some miRNAs may regulate specific individual targets, while others can positively or negatively regulate a variety of cellular processes [11,131]. For example, the balance between oncogenic miRNAs (that target tumor suppressor genes) and tumor suppressive miRNAs (that target oncogenes) may influence tumor development. Sometimes, miRNAs act in concert with transcription factors, creating TF-miRNA transcriptional regulatory networks, such as the p53-miRNA and the NF-κB-miRNA networks that may also interconnect and influence each other [11].

## 4. General Concept: NF-κB Meets miRNAs

NF-κB TFs influence the expression of miRNAs, and importantly NF-κB signaling is also affected by miRNAs which target either the upstream NF-κB activating kinases or other NF-κB signaling components, in positive or negative feedback loops in several different cell types and under different conditions [6,16,52].

### 4.1. MiRNAs Regulated by NF-κB

Several miRNAs, including miR-9, miR-21, miR-30b, miR-143/miR-145, miR-146a, miR-155, miR-221/222, miR-224, miR-301a, and the miR-17-92 cluster have been validated as targets of the NF-κB transcription factors [11,16,52].

Most of these NF-κB-targeted miRNAs have been identified by low throughput methods or unbiased screens. Importantly, the availability of whole-genome data such as transcription factor binding sites based on Chip-Seq experiments, or whole-genome histone modification profiles and also RNA-Seq analyses makes it possible to objectively analyze and efficiently find transcription factors that regulate gene expression. By employing a bioinformatics tool that is used to characterize promoter regions of miRNAs (DIANA miRGen v3.0) [132], we additionally identified 40 miRNAs that contain experimentally verified NF-κB binding sites in their promoter regions (Table 1). Most of these miRNAs are novel potential targets and need further verification. Nevertheless, these data provide an additional, unbiased approach to verify known targets, and also to screen for possible novel targets of specific transcription factors under certain conditions.

Oncogenic miR-21 is an established NF-κB target [42]. NF-κB-dependent induction of miR-21 expression has been detected under different conditions, such as inflammation [16] or DNA damage responses [133] and can target multiple genes, such as *BCL2*, *MASPIN*, *PDCD4*, and *PTEN* [11]. For example, in breast cancer the NF-κB-dependent induction of miR-21 confers chemoresistance and induces cell invasion by repressing *PDCD4* expression which regulates apoptosis, and *PTEN* phosphatase, an inhibitor of Akt pathway that leads to cell survival [133].

In tumor-associated inflammation, the pro-inflammatory cytokine IL-1 leads to NF-κB activation and subsequent upregulation of miR-425 in gastric cancer cells. MiR-425 in turn acts as a tumor promoter by targeting *PTEN* to enhance cell survival [134].

In addition to oncogenic miRNAs, NF-κB can also upregulate tumor suppressive miRNAs, such as miR-143 and miR-145. The expression of these two miRNAs can lead to inhibition of cancer cell proliferation, and also metastasis and invasion by targeting oncogenes such as *MYC*, *ERK5*, and *KRAS*. Non-tumorigenic prostate cells secrete miR-143 to inhibit the growth exclusively of prostate cancer cells that bear activated oncogenes some of which have been mentioned above [11,18].

NF-κB-miR-140 is another regulatory loop. MiR-140 acts as a liver tumor suppressor by negatively regulating NF-κB activity by directly targeting DNA methyltransferase 1 (Dnmt1) expression. In this cellular context, NF-κB suppresses miR-140 expression, resulting in the upregulation of *DNMT1* and increased NF-κB activity, forming a positive feedback loop that promotes liver cancer [135,136]. Aberrant miRNAs have been detected during inflammation and hepatocellular cancer (HCC). Many of these dysregulated miRNAs modulate the initiation and progression of inflammation-induced HCC, the majority of which are NF-κB-regulated miRNAs [137].

Finally, an interesting example of NF-κB-regulated miRNAs is that of miR-221/222, a miRNA family with a dual functional role, acting, in different cellular contexts, either as oncomiRs promoting cancer progression, or as tumor suppressors, promoting cellular senescence [11,12,138,139,140].

### 4.2. NF-κB-Regulating miRNAs

**Figure 1:**
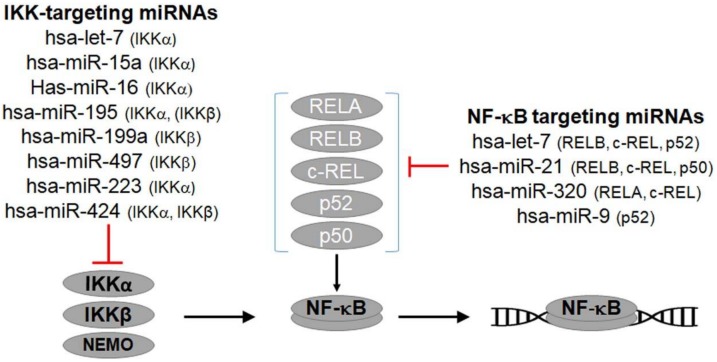
IKK and NF-κB targeting miRNAs. MiRNAs targeting at least one of the NF-κB signaling components such as one of the upstream NF-κB activating kinases, IKKα or IKKβ, or one of the NF-κB transcription factor subunits. For a comprehensive list of NF-κB targeting miRNAs, see Supplementary Table S1.

Multiple miRNAs have been shown to alter NF-κB activity. The current version of Tarbase v8 (http://carolina.imis.athena-innovation.gr/diana_tools/web/index.php?r=tarbasev8%2Findex, access date 20 January 2018), a database comprised of experimentally validated miRNA-gene interactions [141], contains a total of 163 miRNAs that target at least one of the main gene components of NF-κB signaling, either the NF-κB transcription subunits or the upstream NF-κB activating serine/threonine kinases, IKKα and IKKβ (Figure 1 and Supplementary Table S1).

MiR-506 was shown to directly target and downregulate the expression of the NF-κB p65 subunit, leading to the generation of reactive oxygen species (ROS) and the induction of p53-dependent lung cancer cell apoptosis. Interestingly, the p53-dependent induction of miR-506, suggested that miR-506 in lung cancer cells is part of a regulatory network linking p53 and NF-κB signaling [142]. In prostate cancer, the tumor suppressive miR-497 regulates NF-κB signaling by targeting IKKβ, which activates canonical NF-κB signaling leading to inhibition of prostate cancer cell proliferation, migration, and invasion. Importantly, miR-497 expression is reduced in prostate cancer cells, leading to a more aggressive tumor phenotype [143].

The miR-520/373 family has also been shown to act as tumor suppressors in breast cancer, by targeting the RELA/p65 NF-κB subunit. The miR-520/373 family was identified in a genome-wide screen of miRNAs impacting on NF-κB signaling, using a luciferase-based reporter assay in HEK293T cells [144]. This screen identified 13 families of miRNAs, out of which let-7 and miR-181 are known to participate in NF-κB feedback loops [145,146] (discussed in the next section). MiR-520/373 was further analysed and was shown to inhibit NF-κB in estrogen-negative breast cancer cells, which further resulted in downregulation of NF-κB targets such as the pro-inflammatory cytokines IL-6, IL-8, CXCL1, and ICAM-1, leading to the inhibition of tumor-related inflammation, and suppression of tumor growth and metastasis [144]. In another functional screen for miRNAs regulating NF-κB, using a NF-κB reporter cell-line, miR-517a/c were found as potent activators of NF-κB signaling, upregulating the expression of the reporter more than 40-fold. In this case, the identified target of miR-517a/c leading to activation of NF-κB was TNIP1, an inhibitor of NF-κB signaling [147].

MiRNA-126a was shown to target the NF-κB inhibitor, IκBα, leading to canonical NF-κB activation thereby contributing to pathogenesis of ulcerative colitis [148], but paradoxically was shown to act as tumor suppressor for colon cancer [149].

MiRNA-223 was shown to suppress canonical NF-κB signaling in basal keratinocytes to dampen neutrophilic inflammation [150]. MiR-223 limits inflammation and prevents DNA damage and hematological and non-hematological malignancies [151]. MiR-223 is one of the most abundant miRNAs in macrophages and responds to stimuli to control the production of IL-6 and IL-1β [152]. MiR-223 was also associated with macrophage differentiation through targeting IKKα [153]. However, the role of miR-223 in cancer is cell-context dependent [150]. For example, miR-223 promotes the migration and invasion of gastric cancer cells, but has opposite effects in esophageal cancer cells and human cervical cancer [154,155,156].

Several miRNA sites were identified in IKKα including sites for let-7, miR-223, miR-16, and miRNA-142-5p and two target sites for miR15a, one of which overlapped the putative miR-16 site. Further experiments showed that miR15a, miR-16 and miR-223, which target IKKα and are downregulated during macrophage differentiation, they were responsible at least in part for the increase in IKKα protein expression observed during macrophage differentiation [153]. Regulation of IKKα by these miRNAs may contribute to cancer development [157].

MiR-199a negatively regulates the expression of IKKβ in ovarian cancer cells, and inhibits the secretion of pro-inflammatory cytokines, thereby causing suppression of tumor progression and chemoresistance [158]. IKKβ is also targeted by miR-497 in prostate cancer cells and inhibits their cell proliferation, migration, and invasion in vitro [143].

## 5. NF-κB-miRNA Feedback Loops and Transcriptional Regulatory Networks

Multiple feedback loops operating in a specific cell type can act in concert, creating functional networks that control cell fate. There are several NF-κB-miRNA feedback loops in the context of inflammation in normal cells and also during cancer development. These NF-κB-miRNA transcriptional regulatory loops may act in both physiological and pathological conditions, linking pro-inflammatory responses to oncogenic signals [11].

NF-κB signaling during inflammation is self-limiting. A novel feedback loop that has been identified recently involves miR-146a and miR-155, the combinatory action of which controls NF-κB activity during inflammation [18]. Their action is based on a two-step mechanism. First, miR-155 is rapidly upregulated by NF-κB only within the first 12 h of inflammatory response and, by targeting SHIP1, it activates the IKK signalosome complex in a PI3K/Akt-dependent manner, forming a positive feedback loop necessary for signal amplification. Secondly, miR-146a is rather gradually upregulated by NF-κB and forms a negative feedback loop by targeting IRAK1 and TRAF6, ultimately attenuating NF-κB activity in the late phase of inflammation. The combined action of these two positive (NF-κB-miR-155) and negative (NF-κB-miR-146a) NF-κB-miRNA regulatory loops provides optimal NF-κB activity during inflammatory stimuli, and eventually lead to the resolution of the inflammatory response [18].

Knockout of miR-146a in C57BL/6 mice leads to myeloid sarcomas and some lymphomas, and the animals exhibit chronic myeloproliferation in their bone marrow. The development of myeloid malignancies correlated with increased canonical NF-κB activity. Genetic ablation of NF-κB p50 suppressed myeloproliferation suggesting that NF-κB was required for myeloproliferative disease [159].

MiR-9 is induced by pro-inflammatory signals in a NF-κB-dependent manner in human monocytes [160]. MiR-9 targets the *NFKB1* gene, which encodes the p105/p50 precursor subunit and renders lung cancer cells sensitive to ionizing radiation [160]. In ovarian cancer, miR-9 also targets *NFKB1* and its downregulation in this cancer type, as compared to normal ovarian tissue is considered an additional tumor-promoting mechanism [161]. The fact that miR-9 is positively regulated by inflammation-induced canonical NF-κB (RelA/65-p50) signaling, taken together with the finding that miR-9 targets *NFKB1* (p105/p50), suggests a negative feedback loop mechanism fine tuning the inflammatory response with an impact in cancer.

Another negative feedback-loop in acute myeloid leukemia (AML), bearing *KIT* driver mutations, involves miR-29b and NF-κB. MiR-29b targets the Sp1 transcription factor. In *KIT*-driven AML, *KIT* upregulates Sp1, which in turn binds NF-κB and transactivates *KIT*. *Sp1* escapes from miR-29b downregulation through a negative feedback loop, in which Sp1-induced NF-κB recruits HDACs in the miR-29b promoter leading to its transcriptional repression [162].

A positive feedback loop that keeps NF-κB in an activated state operates in breast cancer cells after chemotherapy. In these cells, chemotherapy activates NF-κB which targets and downregulates miR-448 by binding to its promoter, leading to increased expression of the miR-448 target special AT-rich sequence-binding protein-1 (SATB1). SATB1 upregulation ultimately leads to Twist1 expression, a regulator of EMT; and it also further enhances NF-κB activity, forming a positive feedback loop that simultaneously promotes EMT [163].

One of the most well-defined regulatory networks that link inflammation and cancer has been extensively studied by Iliopoulos et al. and is formed by two distinct and complimentary feedback-loops involving either NF-κB, Lin28, let-7 miRNA and IL-6 or IL-6, miR-21, and miR-181b-1 miRNAs, *PTEN,* CYLD, and NF-κB [145,146]. During oncogenesis, proinflammatory signals that are mediated by NF-κB, upregulate Lin28, which downregulates the tumor suppressor let-7 miRNA [164] which targets IL-6. Let-7 downregulation results in increased IL-6 levels, further activating NF-κB, generating a feedback loop that sustains inflammation and promotes oncogenesis [145]. NF-κB can also remain active by a complimentary feedback-loop that involves miR-21 and miR-181b-1. IL-6 activates STAT3, an inducer of miR-21 and miR-181b-1 expression, which respectively target *PTEN* and *CYLD*. *PTEN* and CYLD inhibition further leads to NF-κB activation [146]. Therefore, the combined action of NF-κB and STAT3 leading to the induction of miR-21 and miR-181b-1 and let-7 downregulation, ultimately act as a feedback mechanism linking inflammation to cancer. In addition to NF-κB, STAT3 can also be further upregulated as a result of this feedback mechanism, since miR-181a/b induction by STAT3 can also activate the IL-6/STAT3 signaling pathway [146]. More recently, studies on the interplay between NF-κB and STAT3, two of the main transcription factors that regulate inflammation [44,45,46] have revealed that feedback mechanisms that involve these two factors also include several miRNAs [165]. Studies revealed the existence of a negative feedback loop mechanism between STAT3 and NF-κB involving miR-146b. In this mechanism, STAT3 targets miR-146b, which downregulates NF-κB, reducing IL-6 production. The reduction of IL-6 is the final step of a negative feedback loop, since IL-6 activates STAT3, contributing to chronic inflammation. This is also a mechanism linking inflammation and cancer in breast tissue, whereas in normal tissue miR-146b is upregulated, leading to resolution of inflammation, in breast cancer it is downregulated, leading to chronic inflammation, through deregulation of the above feedback loop and cancer development [166].

A constitutively activated feedforward circuit composed of IκBα/NF-κB(p65) and miR-196b-3p, was shown to drive castration-resistant prostate cancer (CRPC) development. Constitutive activation of IκBα/RelA(p65) in this circuit was independent of the activation of the canonical IKKβ/NF-κB pathway [167].

The availability of genomic data makes it possible to improve our knowledge of novel regulatory networks that exist in physiological or pathological conditions. Using bioinformatics tools and analysis we were able to identify candidate miRNAs regulated by NF-κB (Table 1) or targeting NF-κB pathway components (Figure 1 and Supplementary Table S1). Another such tool that offers a pathway-based approach, is the server of Diana miRpath for finding specific miRNAs involved in pathways or regulatory networks [168]. We believe that the exploitation of unbiased genomic data in conjunction with experimental validation may confirm biologically relevant findings and relate them to specific functions and (physiological or pathological) conditions.

## 6. Final Thoughts: Possible Therapeutic Approaches

In the current review, we focused on the interplay between NF-κB and miRNAs impacting on inflammation and cancer development. The functional role of miRNAs in these processes is due to their action as epigenetic switches that interconnect signaling pathways and cellular processes, integrating in larger regulatory networks. In this conceptual framework, the expression of miRNAs may offer the possibility to: (a) fine-tune the activity of a process in time, such as the expression of miR-155 and miR-146b regulating NF-κB expression and inflammation intensity and duration [18]; (b) amplify or attenuate the activity of a signaling pathway, by taking part in feedback-loops, such as the NF-κB-miRNA amplifying loops in inflammation linked to cancer [145,146]; and (c) interconnect TF-miRNA opposing regulatory pathways such as the p53-miRNA and NF-κB-miRNA networks. Certain NF-κB-regulated miRNAs can regulate p53, and vice versa, hence they can shift the balance towards apoptosis or cell survival and determine the fate of a cancer cell [11]. The complexity of epigenetic regulation requires taking into account aspects such as the expression of specific TFs and miRNAs and their possible interconnection.

Based on the dynamic nature of NF-κB signaling combined with the diverse actions and multiple targets of miRNAs, we believe that the NF-κB-miRNA feedback regulatory loop mechanisms discussed above or possibly novel ones yet to be discovered, should be considered when studying inflammatory responses linked to cancer initiation, progression, and development. Understanding of the NF-κB-miRNA transcription factor regulatory networks may offer opportunities for pharmacological exploitation and personalized treatments.

## Figures and Tables

**Table 1:** MiRNAs containing experimentally verified NF-κB binding sites in their promoter (miRGen v3.0 tool).

miRNA Name	Chromosomal Location of Promoter (hg19)	Strand
hsa-let-7a-1	chr9:96929483–96929484	[+]
hsa-let-7d	chr9:96929483–96929484	[+]
hsa-let-7f-1	chr9:96929483–96929484	[+]
hsa-let-7i	chr12:62997400–62997401	[+]
hsa-mir-101-1	chr1:65532138–65532139	[−]
hsa-mir-1204	chr8:128806768–128806769	[+]
hsa-mir-1205	chr8:128806768–128806769	[+]
hsa-mir-1206	chr8:128806768–128806769	[+]
hsa-mir-1207	chr8:128806759–128806760	[+]
hsa-mir-1208	chr8:128806759–128806760	[+]
hsa-mir-124-1	chr8:9763203–9763204	[−]
hsa-mir-125b-1	chr11:121971206–121971207	[−]
hsa-mir-1289-1	chr20:34042503–34042504	[−]
hsa-mir-135b	chr1:205426509–205426510	[−]
hsa-mir-137	chr1:98520169–98520170	[−]
hsa-mir-146a	chr5:159894835–159894836	[+]
hsa-mir-148a	chr7:25990290–25990291	[−]
hsa-mir-193a	chr17:29886484–29886485	[+]
hsa-mir-22	chr17:1618561–1618562	[−]
hsa-mir-223	chrX:65219544–65219545	[+]
hsa-mir-23a	chr19:13953455–13953456	[−]
hsa-mir-24-2	chr19:13953455–13953456	[−]
hsa-mir-2682	chr1:98520169–98520170	[−]
hsa-mir-27a	chr19:13953455–13953456	[−]
hsa-mir-2861	chr9:130548069–130548070	[+]
hsa-mir-29a	chr7:130794752–130794753	[−]
hsa-mir-29b-1	chr7:130794752–130794753	[−]
hsa-mir-30a	chr6:72130555–72130556	[−]
hsa-mir-30c-2	chr6:72130555–72130556	[−]
hsa-mir-3142	chr5:159894835–159894836	[+]
hsa-mir-3199-2	chr22:28315414–28315415	[+]
hsa-mir-365b	chr17:29886484–29886485	[+]
hsa-mir-3667	chr22:50051180–50051181	[−]
hsa-mir-3672	chrX:120325891–120325892	[+]
hsa-mir-3679	chr2:134877461–134877462	[+]
hsa-mir-3960	chr9:130548069–130548070	[+]
hsa-mir-4725	chr17:29886484–29886485	[+]
hsa-mir-505	chrX:139015225–139015226	[−]
hsa-mir-5194	chr8:131028942–131028943	[−]
hsa-mir-612	chr11:65190256–65190257	[+]

## Supplementary Materials

The following are available online at http://www.mdpi.com/2227-9059/6/2/40/s1, Table S1: Validated miRNAs for targeting at least one of NF-κB signaling components. (# Denotes the number of NF-κB genes targeted).
