# Supplementary material for: Roles of NF-κB Signaling in the Regulation of miRNAs Impacting on Inflammation in Cancer
Source: Biomedicines. 2018 Mar 29;6(2):40. doi: 10.3390/biomedicines6020040 (PMC6027290; doi:10.3390/biomedicines6020040)
Supplement: Supplementary file 1 [file biomedicines-06-00040-s001.pdf]

Supplementary Table 1. Validated miRNAs for targeting at least one of NF-κB signaling components. (# Denotes the number of NF- κB genes targeted).

miRNA name	#	miRNA name	#
hsa-let-7a-5p	2	hsa-miR-16-5p	7
hsa-let-7b-5p	4	hsa-miR-17-5p	1
hsa-let-7c-5p	2	hsa-miR-181a-5p	1
hsa-let-7e-5p	1	hsa-miR-181c-5p	1
hsa-let-7f-5p	1	hsa-miR-181d-5p	1
hsa-let-7g-5p	1	hsa-miR-182-5p	2
hsa-let-7i-5p	1	hsa-miR-185-3p	1
hsa-miR-101-3p	1	hsa-miR-186-5p	1
hsa-miR-103a-3p	2	hsa-miR-191-5p	5
hsa-miR-106b-3p	1	hsa-miR-193b-3p	1
hsa-miR-107	2	hsa-miR-195-5p	2
hsa-miR-10a-3p	1	hsa-miR-196a-5p	1
hsa-miR-10a-5p	1	hsa-miR-198	1
hsa-miR-1179	1	hsa-miR-199a-5p	2
hsa-miR-1224-5p	1	hsa-miR-200a-3p	1
hsa-miR-1229-3p	1	hsa-miR-200b-3p	1
hsa-miR-124-3p	3	hsa-miR-200c-3p	2
hsa-miR-124-5p	1	hsa-miR-205-5p	1
hsa-miR-1263	1	hsa-miR-210-3p	1
hsa-miR-126-5p	1	hsa-miR-2115-5p	1
hsa-miR-1268a	1	hsa-miR-21-3p	2
hsa-miR-1268b	1	hsa-miR-218-1-3p	1
hsa-miR-1271-5p	1	hsa-miR-219a-5p	2
hsa-miR-1299	1	hsa-miR-223-3p	1
hsa-miR-130a-3p	3	hsa-miR-22-3p	2
hsa-miR-130b-3p	2	hsa-miR-23a-3p	1
hsa-miR-132-3p	1	hsa-miR-23b-3p	2
hsa-miR-135a-5p	1	hsa-miR-23c	1
hsa-miR-135b-5p	1	hsa-miR-24-3p	1
hsa-miR-139-5p	1	hsa-miR-25-5p	1
hsa-miR-1-3p	2	hsa-miR-26a-5p	1
hsa-miR-141-3p	2	hsa-miR-26b-3p	1
hsa-miR-146a-5p	2	hsa-miR-26b-5p	2
hsa-miR-146b-5p	1	hsa-miR-27a-3p	3
hsa-miR-147a	1	hsa-miR-27a-5p	3
hsa-miR-148a-3p	2	hsa-miR-27b-3p	1
hsa-miR-148b-3p	1	hsa-miR-28-5p	1
hsa-miR-152-3p	1	hsa-miR-29a-3p	2
hsa-miR-155-5p	6	hsa-miR-29b-3p	2
hsa-miR-15a-5p	2	hsa-miR-29c-3p	2
hsa-miR-15b-5p	2	hsa-miR-301a-3p	2
hsa-miR-301b-3p	2	hsa-miR-503-5p	1
hsa-miR-30a-3p	3	hsa-miR-513a-5p	1
hsa-miR-30a-5p	1	hsa-miR-520c-3p	1
hsa-miR-30c-1-3p	1	hsa-miR-520d-5p	1
hsa-miR-30d-5p	1	hsa-miR-522-5p	1
hsa-miR-30e-3p	3	hsa-miR-532-3p	2
hsa-miR-30e-5p	1	hsa-miR-532-5p	1
hsa-miR-31-5p	1	hsa-miR-545-3p	1
hsa-miR-320a	1	hsa-miR-548an	1
hsa-miR-320b	1	hsa-miR-548d-3p	2
hsa-miR-320c	1	hsa-miR-548h-3p	1

hsa-miR-320d	1	hsa-miR-548z	1
hsa-miR-320e	1	hsa-miR-5699-3p	1
hsa-miR-335-5p	1	hsa-miR-574-5p	1
hsa-miR-338-3p	1	hsa-miR-576-5p	1
hsa-miR-34a-3p	1	hsa-miR-589-5p	2
hsa-miR-34a-5p	2	hsa-miR-590-3p	1
hsa-miR-3613-3p	1	hsa-miR-625-5p	2
hsa-miR-365a-3p	1	hsa-miR-628-3p	1
hsa-miR-365b-3p	1	hsa-miR-628-5p	1
hsa-miR-3679-5p	1	hsa-miR-629-3p	1
hsa-miR-3682-3p	1	hsa-miR-635	1
hsa-miR-372-3p	1	hsa-miR-641	1
hsa-miR-373-3p	1	hsa-miR-642a-5p	1
hsa-miR-374a-5p	1	hsa-miR-643	1
hsa-miR-374b-3p	1	hsa-miR-652-3p	1
hsa-miR-375	1	hsa-miR-653-5p	1
hsa-miR-376a-5p	1	hsa-miR-675-5p	1
hsa-miR-424-5p	2	hsa-miR-7-1-3p	1
hsa-miR-431-3p	1	hsa-miR-7-2-3p	1
hsa-miR-4317	1	hsa-miR-7-5p	4
hsa-miR-449a	1	hsa-miR-769-3p	1
hsa-miR-4511	1	hsa-miR-873-5p	1
hsa-miR-452-5p	1	hsa-miR-874-3p	1
hsa-miR-4685-3p	1	hsa-miR-922	2
hsa-miR-4726-3p	1	hsa-miR-92a-1-5p	1
hsa-miR-4786-3p	1	hsa-miR-939-5p	1
hsa-miR-497-5p	2	hsa-miR-9-5p	1
hsa-miR-4999-5p	1	hsa-miR-96-5p	1
hsa-miR-500a-5p	1	hsa-miR-98-5p	3
